# Effects of balance training on balance performance in youth: role of training difficulty

**DOI:** 10.1186/s13102-020-00218-4

**Published:** 2020-11-23

**Authors:** Simon Schedler, Florian Tenelsen, Laura Wich, Thomas Muehlbauer

**Affiliations:** Division of Movement and Training Sciences/Biomechanics of Sport, University of Duisburg-Essen, Gladbecker Str. 182, 45141 Essen, Germany

**Keywords:** Postural control, Adolescence, Intervention, Dose-response relationship

## Abstract

**Background:**

Cross-sectional studies have shown that balance performance can be challenged by the level of task difficulty (e.g., varying stance conditions, sensory manipulations). However, it remains unclear whether the application of different levels of task difficulty during balance training (BT) leads to altered adaptations in balance performance. Thus, we examined the effects of BT conducted under a high versus a low level of task difficulty on balance performance.

**Methods:**

Forty male adolescents were randomly assigned to a BT program using a low (BT-low: *n* = 20; age: 12.4 ± 2.0 yrs) or a high (BT-high: *n* = 20; age: 12.5 ± 2.5 yrs) level of balance task difficulty. Both groups trained for 7 weeks (2 sessions/week, 30–35 min each). Pre- and post-training assessments included measures of static (one-legged stance [OLS] time), dynamic (10-m gait velocity), and proactive (Y-Balance Test [YBT] reach distance, Functional Reach Test [FRT]; Timed-Up-and-Go Test [TUG]) balance.

**Results:**

Significant main effects of Test (i.e., pre- to post-test improvements) were observed for all but one balance measure (i.e., 10-m gait velocity). Additionally, a Test x Group interaction was detected for the FRT in favor of the BT-high group (Δ + 8%, *p* < 0.001, *d* = 0.35). Further, tendencies toward significant Test x Group interactions were found for the YBT anterior reach (in favor of BT-high: Δ + 9%, *p* < 0.001, *d* = 0.60) and for the OLS with eyes opened and on firm surface (in favor of BT-low: Δ + 31%, *p* = 0.003, *d* = 0.67).

**Conclusions:**

Following 7 weeks of BT, enhancements in measures of static, dynamic, and proactive balance were observed in the BT-high and BT-low groups. However, BT-high appears to be more effective for increasing measures of proactive balance, whereas BT-low seems to be more effective for improving proxies of static balance.

**Trial registration:**

Current Controlled Trials ISRCTN83638708 (Retrospectively registered 19th June, 2020).

## Background

The effectiveness of balance training (BT) for improving different components of balance performance in children and adolescents has been shown by several original studies [1–4] and these findings have been summarized in systematic reviews [5, 6]. Contrary, recommendations on how to design BT with respect to different load dimensions (e.g., training volume, training intensity) in order to be most effective in children and adolescents are rather unspecific and have only been derived from review articles [5, 7]. For example, a reduction in the base of support / sensory input and the inclusion of unstable surfaces / cognitive and motor interference tasks have been proposed as being effective means to increase task difficulty and thus enhance balance performance in youth [7]. However, although these recommendations seem to be justified based on the available literature, empirical evidence is still lacking. Indeed, Gebel et al. [5] conducted a systematic review with meta-analysis analyzing the effects and dose-response relationships of BT on balance performance in youth. Yet, using this approach dose-response relationships are compared indirectly instead of directly. In other words, results from a study with a short intervention period were for instance compared to those from a study with a long one, but there was no comparison of the effects of different intervention periods within a single study. Additionally, dose-response relationships of BT in youth could only be quantified for certain training modalities (i.e., training period, training frequency). To summarize, there is a need for research which (i) directly compares different training modalities within a single study and (ii) investigates load dimensions that have not been analyzed so far.

One of these overlooked load dimensions is „training difficulty”. This is especially surprising as changing the level of balance task demand (e.g., varying stance conditions or manipulating the sensory input) has been described as being one way to design varied and challenging BT [7, 8]. Further, cross-sectional studies [9–13] showed that balance performance can be challenged by the level of task difficulty. In this regard, Muehlbauer et al. [10] reported an increase in postural sway when the base of support was reduced (i.e., from bipedal stance over step and tandem stance to unipedal stance) and the sensory input was altered (i.e., from standing with eyes opened on firm ground over eyes opened on foam ground to eyes closed on firm ground). However, the effects of conducting BT with different balance task demands on balance performance in youth have not been investigated in the context of regular BT lasting several weeks.

Thus, the aim of the present study was to investigate the effects of BT conducted under a high versus a low level of task difficulty on balance performance in youth. We expected that both exercise conditions will result in enhanced balance performance but the improvement will be larger in the group that used a high compared to a low level of balance task difficulty during training.

## Methods

### Participants

**Fig. 1:**
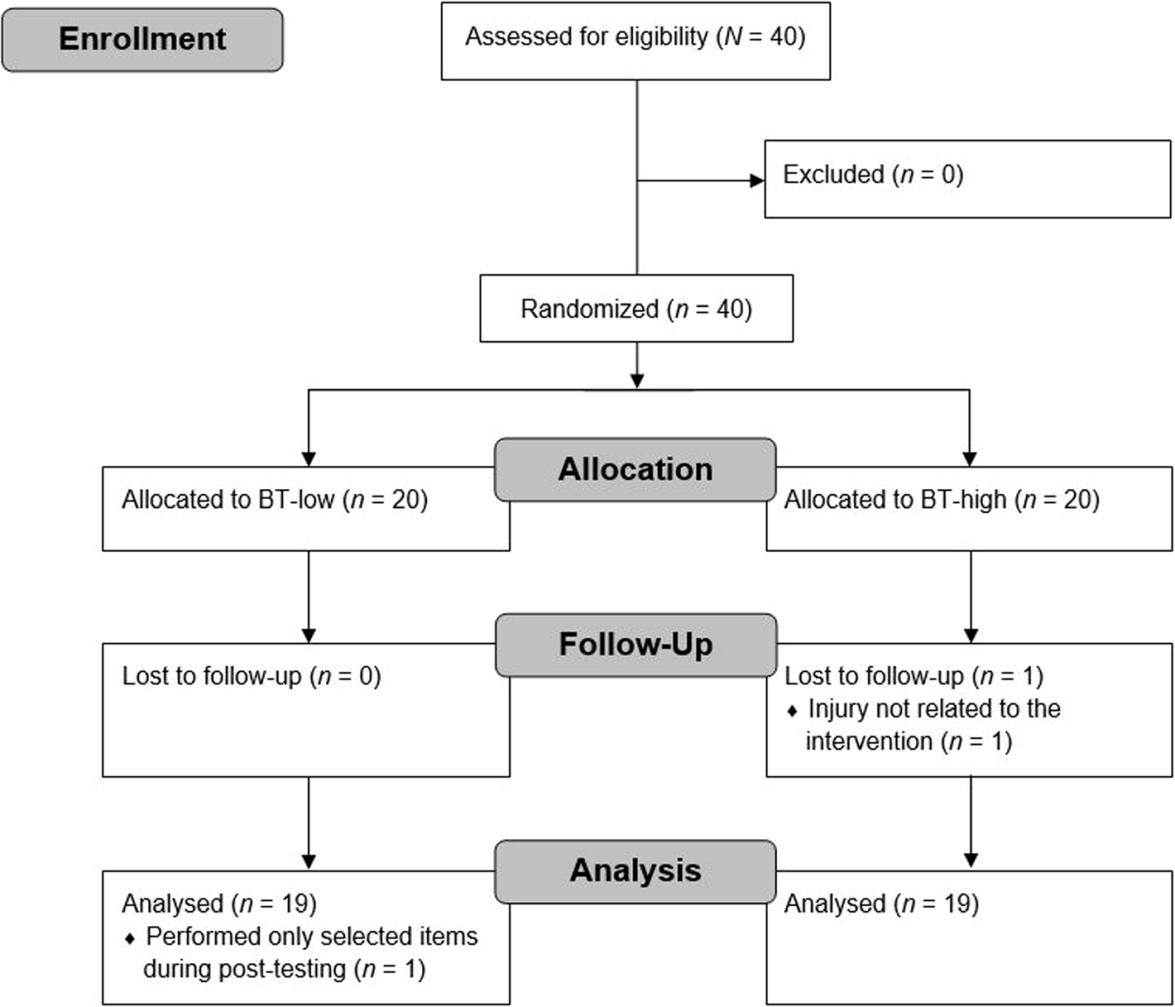
Flow diagram of the progress through the phases of the study according to the CONSORT statements. BT-low = balance training using a low level of task difficulty; BT-high = balance training using a high level of task difficulty

Using G*Power [14], the power analysis (*f* = 0.25, α = 0.05, 1-β = 0.80, number of groups: *n* = 2, number of measurements: *n* = 2, correlation between testing: *r* = 0.50, drop-out rate per group: 10% due to injury reasons not attributable to treatments) revealed that a total sample size of *N* = 37 participants (i.e., *n* = 18–19 per group) would be sufficient to detect statistically significant training effects. Therefore, forty healthy male adolescents from a local sports club were assessed for eligibility and randomly assigned to either a BT-low (*n* = 20; age: 12.4 ± 2.0 years, body mass: 61.0 ± 22.6 kg, body height: 159.0 ± 13.7 cm; body mass index: 23.9 ± 7.7 kg/m^2^) or a BT-high (*n* = 20; age: 12.5 ± 2.5 years, body mass: 64.5 ± 22.1 kg, body height: 162.4 ± 14.2 cm; body mass index: 24.0 ± 6.5 kg/m^2^) group using Research Randomizer (www.randomizer.org) (Fig. 1). Participants were free of any known neurological, musculoskeletal, or orthopedic diseases. Over the course of the study, a participant of the BT-high group dropped out due to an injury not related to the intervention. In addition, one subject of the BT-low group performed only selected items during post-testing and was thus excluded from statistical analyses. The study was conducted in accordance with the CONSORT guidelines [15]. Participant recruitment began in January 2019 and the assessments and intervention were carried out the following months. Participants’ assent and parents’ written informed consent were obtained prior to the start of the study. The Human Ethics Committee at the University of Duisburg-Essen, Faculty of Educational Sciences approved the study protocol (approval code: TM_06_12_2018).

### Testing procedures

**Fig. 2:**
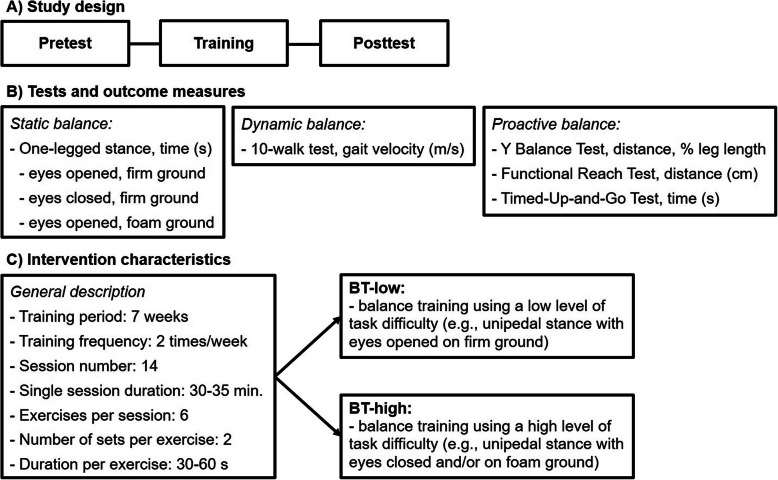
Schematic description of the study design (**a**), tests and outcome measures (**b**), and the intervention characteristics (**c**). BT-low = balance training using a low level of task difficulty; BT-high = balance training using a high level of task difficulty

The pre- and post-testing was conducted in a gym hall by the same skilled assessors (graduated sport scientists) before and after the 7-weeks training period (Fig. 2a). All participants received standardized verbal instructions and a visual demonstration regarding the testing procedure that included assessment of anthropometric variables, static, dynamic and proactive balance (Fig. 2b). One practice followed by one data-registration trial was performed for each test, unless otherwise stated. All subject conducted a standardized 10-min warm-up prior to each testing that consisted of submaximal running (e.g., skipping, hip in/out) and balance exercises (e.g., forward/backward beam walking, single leg stance on unstable devices).

#### Assessment of static balance

For the assessment of static balance, the participants were asked to stand without shoes on their non-dominant leg (determined per self-report), hands placed on hips and gaze fixed on a cross on the nearby wall. The participants were instructed to perform the one-legged stance as long as possible but for a maximum of 60 s. The maximal stance time (s) was used for further analysis. Static balance performance was assessed under three different conditions: (1) standing with eyes opened on firm ground; (2) standing with eyes closed on firm ground; (3) standing with eyes opened on foam ground (i.e., AIREX Balance-pad).

#### Assessment of dynamic balance

Dynamic balance was assessed using the 10-m walk test. The participants were instructed to walk with their preferred speed, initiating and terminating the walk a minimum of one meter before and after the 10-m walkway. The time needed to perform the test was measured with a stopwatch to the nearest 0.01 s. Gait speed (m/s) was calculated as the 10-m walking distance (m) divided by walking time (s) and used for further analysis.

#### Assessment of proactive balance

Proactive balance was assessed by means of the Y-Balance Test (YBT) Kit (Functional Movement Systems®, Chatham, USA). The test kit consists of a centralized stance platform to which three pipes were attached that represent the anterior (AT), posteromedial (PM), and posterolateral (PL) reach directions. Each pipe is marked in 1.0-cm increments for measurement purposes and equipped with a moveable reach indicator. Before the YBT began, the respective lengths of the participants’ non-dominant leg was determined in supine position by measuring the distance from the anterior superior iliac spine to the most distal aspect of the medial malleolus [16]. Afterwards, participants were asked to reach with the dominant leg as far as possible in the AT, PM, and PL directions while standing with their non-dominant leg on the centralized stance platform. A total of six trials (three practice trials followed by three data-collection trials) were executed. The maximal reach distance (cm) per reach direction was used for further analysis In this regard, the normalized maximal reach distance (% leg length [LL]) per reach direction was calculated by dividing the absolute maximal reach distance (cm) by LL (cm) and then multiplying by 100. In addition, the normalized (% LL) composite score (CS) was computed as the sum of the absolute maximal reach distance (cm) per reach direction divided by three times LL (cm) and then multiplied by 100 and used for analysis as well.

Proactive balance was further assessed using the Functional Reach Test (FRT) [17]. The participants’ task was to reach with the non-dominant arm (determined by self-report) forward while maintaining a fixed base of support in the standing position. Maximal reach distance (cm) was determined with a measuring tape that was fixated on a wall. Two trials with a 60-s break between trials were performed and the best trial (i.e., largest distance) was used for further analysis.

The Timed-Up-and-Go Test (TUG) was additionally used for the assessment of proactive balance performance [18]. In this regard, the participants were asked to rise from a chair, walk three meters, turn around, walk back to the chair and sit down. The time (s) needed to perform the TUG was recorded with a stopwatch to the nearest 0.01 s. Each participant performed two trials with 60 s in between and the best trial (i.e., shortest time) was used for further analysis.

### Balance training programs

Both groups conducted 7 weeks of BT (2 sessions/week, 30–35 min each) that included six balance exercises per training session (Fig. 2c). After a 15-min warm-up program including general (e.g., skipping, running with hip in/out) and specific (e.g., two−/one-legged stance on unstable devices, forward/backward beam walking) exercises, two sets per balance exercise were performed for 30 s each with a 60 s rest period between sets and a 90 s break between exercises. While participants of the BT-low group performed all balance exercises with a low level of task difficulty, subjects in the BT-high group executed the same or similar exercises using a high level of task difficulty (Table 1). Yet, both groups executed the same training volume (i.e., number of exercises, number of sets per exercise, and duration per set of exercise). Progression during training was achieved by means of increasing exercise duration (i.e., from 30 s over 45 s to 60 s), change of stance (i.e., two-legged stance, tandem stance, one-legged stance) and walking (i.e., forward, backward) condition, manipulation of visual input (e.g., eyes opened vs. closed), and concurrent execution of cognitive (i.e., backward counting) or motor (i.e., throwing/catching a ball) tasks. All training sessions ended with a 15-min cool-down that included flexibility exercises and jogging at light intensity.

**Table 1:** Description of the balance training programs by the level of task difficulty

Group	Week 1 (session 1 & 2)	Week 2 (session 3 & 4)	Week 3 (session 5 & 6)	Week 4 (session 7 & 8)	Week 5 (session 9 & 10)	Week 6 (session 11 & 12)	Week 7 (session 13 & 14)
BT-low	a) Tandem stance: eyes opened, with arm support on a balance pad b) Two-legged stance: eyes opened, with arm support on an ankle disc c) Two-legged stance: eyes opened, with arm support on a balance board (difficulty level 1) d) One-legged stance: eyes opened, with arm support on an air cushion e) Walking forward: eyes opened, with arm support f) Tandem walking forward: between two lines, eyes opened, with arm support	a) One-legged stance: eyes opened, with arm support on a balance pad b)–e) cf. week 1 but without arm support f) Tandem walking forward: on a line, eyes opened, with arm support	a) cf. week 2 b) Two-legged stance: squatting position, eyes opened, with arm support on an ankle disc c) Two-legged stance: eyes opened, with arm support on a balance board (difficulty level 2) d) Walking backward: eyes opened, with arm support on a balance beam e) One-legged stance: change from eyes opened to close every 5 s f) Flamingo stance: eyes opened, with arm support	a) Two-legged stance: squatting position, eyes opened, with arm support on a balance pad b) cf. week 3 c) Two-legged stance: eyes opened, with arm support on a balance board (difficulty level 3) d) cf. week 3 e) Two-legged stance: squatting position, eyes opened, with arm support on an air cushion f) Flamingo stance: eyes opened, without arm support	a) Two-legged stance: squats, eyes opened, without arm support on a balance pad b) One-legged stance: eyes opened, with arm support on an ankle disc c) Two-legged stance: squatting position, eyes opened, with arm support on a balance board (difficulty level 3) d) Walking backwards: eyes closed, without arm support on a balance beam e) Two-legged stance: squats, eyes opened, with arm support on a sissle f) One-legged stance: squats, eyes opened, without arm support	a) One-legged stance: eyes closed, with arm support on a balance pad b) Two-legged stance: squats, eyes opened, with arm support on an ankle disc c) Two-legged stance: squats, eyes opened, with arm support on a balance board (difficulty level 3) d) Walking backwards: eyes opened, with arm support on a balance beam, counting backwards e) Flamingo stance: eyes opened, with arm support on a sissle f) Y Balance test reaches, eyes opened, with arm support	a) One-legged stance: eyes closed, without arm support on a balance pad b) cf. week 6 c) Two-legged stance: squats, eyes closed, with arm support on a balance board (difficulty level 3) d) Walking backwards: eyes closed, with arm support on a balance beam, counting backwards e) One-legged stance: squats, eyes opened, with arm support f) Y Balance test reaches, eyes closed, with arm support
BT-high	a) Tandem stance, **eyes closed**, with arm support on a balance pad b) Two-legged stance: **eyes closed**, with arm support on an ankle disc c) Two-legged stance: eyes opened, with arm support on a balance board (**difficulty level 4**) d) One-legged stance: **eyes closed**, with arm support on an air cushion e) Walking forward: eyes opened, with arm support on a **balance beam** f) Tandem walking forward: between two lines, eyes opened, **without arm support**	a) One-legged stance: **eyes closed**, with arm support on a balance pad b)–e) cf. week 1 but without arm support f) Tandem walking forward: on a line, eyes opened, **without arm support**	a) cf. week 2 b) Two-legged stance: squatting position, **eyes closed**, with arm support on an ankle disc c) Two-legged stance: **eyes closed**, with arm support on a balance board (**difficulty level 4**) d) **Walking forward**: eyes opened, with arm support on a balance beam, **counting backwards** e) **Tandem stance: eyes closed, counting backwards** f) Flamingo stance: **eyes closed**, with arm support	a) Two-legged stance: squatting position, **eyes closed**, with arm support on a balance pad b) cf. week 3 c) Two-legged stance: eyes opened, **without arm support** on a balance board (difficulty level 4) d) cf. week 3 e) Two-legged stance: squatting position, **eyes closed**, with arm support on an air cushion f) Flamingo stance: **eyes closed**, without arm support	a) Two-legged stance: squats, **eyes closed**, without arm support on a balance pad b) One-legged stance: eyes opened, **without arm support** on an ankle disc c) Two-legged stance: squatting position, eyes opened, with arm support on a balance board (**difficulty level 5**) d) Walking backwards**: eyes closed**, without arm support on a balance beam, **counting backwards** e) Two-legged stance: squats, **eyes closed**, with arm support on an air cushion f) One-legged stance: squats, **eyes closed**, without arm support	a) One-legged stance: eyes closed, with arm support on a balance pad, **counting backwards** b) Two-legged stance: squats, **eyes closed**, with arm support on an ankle disc c) Two-legged stance: squats, eyes opened, with arm support on a balance board (**difficulty level 6**) d) Walking backwards: eyes opened, on a balance beam and **with basketball dribbling** e) Flamingo stance: eyes opened, **without arm support** on an air cushion f) Y Balance test reaches, eyes opened, **without arm support**	a) One-legged stance: eyes closed, without arm support on a balance pad, **counting backwards** b) cf. week 6 c) Two-legged stance: squats, eyes closed, with arm support on a balance board (**difficulty level 6**) d) Walking backwards: eyes opened, on a balance beam and **with throwing/catching a tennis ball** e) One-legged stance: squats, eyes opened, **without arm support** f) Y Balance test reaches, eyes closed, **without arm support**

Bold font indicates differences in task difficulty. The balance board (Wobblesmart©, Artzt GmbH, Dornburg, Germany) is equipped with a mechanically adjustable pivot to increase task difficulty from level 1 (low) to 6 (high). *BT-low* Balance training using a low level of task difficulty, *BT-high* Balance training using a high level of task difficulty

### Statistical analyses

Descriptive data are reported as group mean values and standard deviations. An univariate analysis of variance (ANOVA) was conducted to test for significant differences in pre-testing values between the two groups. Thereafter, a 2 (Test: pre, post) × 2 (Group: BT-high, BT-low) ANOVA with repeated measures on Test was used. In the case of a significant (*p* < 0.05) or a tendency toward a significant (.05 ≤ *p* < 0.10) Test × Group interaction, differences between pre- and post-testing values were analyzed for each group separately using paired *t*-tests. Further, effect sizes were calculated by converting partial eta-squared to Cohen’s *d*. In accordance to Cohen [19], 0 ≤ *d* ≤ 0.49 represent small effects, 0.50 ≤ *d* ≤ 0.79 represent moderate effects, and *d* ≥ 0.80 represent large effects. All statistical analyses were performed using Statistical Package for Social Sciences version 24.0.

## Results

Table 2 displays statistics for all analyzed variables. In general, there were no statistically significant differences in pre-test values between the two intervention groups. Further, the attendance rates during training sessions amounted to 93.5 and 93.0% in the BT-high and BT-low group, respectively.

**Table 2:** Effects of balance training using a high versus low level of task difficulty on measures of balance in youth

Variables	BT-low (*n* = 19)			BT-high (*n* = 19)			*p*-value (Cohen’s *d*)		
	Pre	Post	∆%^a^	Pre	Post	∆%^a^	Test	Test x Group	Group
*Static balance*									
OLS time; EO, FI [s]	44.8 ± 19.8	58.7 ± 10.3	+ 31	50.4 ± 16.1	54.9 ± 13.9	+ 9	.001 (1.22)	.078 (0.61)	.798 (0.09)
OLS time; EC, FI [s]	13.4 ± 11.7	30.0 ± 17.8	+ 124	23.7 ± 19.2	40.8 ± 20.8	+ 72	<.001 (1.83)	.935 (0.04)	.037 (0.72)
OLS time; EO, FO [s]	23.7 ± 18.5	49.6 ± 17.5	+ 109	31.1 ± 21.6	49.4 ± 17.4	+ 59	<.001 (2.03)	.301 (0.35)	.466 (0.25)
*Dynamic balance*									
Gait velocity [m/s]	1.63 ± 0.26	1.66 ± 0.22	+ 2	1.71 ± 0.28	1.69 ± 0.20	−1	.909 (0.05)	.387 (0.29)	.461 (0.25)
*Proactive balance*									
AT [% LL]	69.8 ± 9.2	73.2 ± 9.7	+ 5	71.6 ± 10.4	78.1 ± 11.1	+ 9	<.001 (1.96)	.074 (0.61)	.301 (0.35)
PM [% LL]	110.3 ± 9.6	112.5 ± 7.7	+ 2	109.6 ± 13.9	114.9 ± 10.8	+ 5	.001 (1.26)	.125 (0.52)	.802 (0.09)
PL [% LL]	104.4 ± 11.1	109.1 ± 7.4	+ 5	107.9 ± 12.8	113.1 ± 10.6	+ 5	<.001 (1.51)	.829 (0.06)	.253 (0.39)
CS [% LL]	94.9 ± 8.7	98.3 ± 7.0	+ 4	96.4 ± 11.1	102.1 ± 9.2	+ 6	<.001 (2.15)	.110 (0.54)	.356 (0.31)
FRT [cm]	42.7 ± 7.2	43.6 ± 6.4	+ 2	42.4 ± 9.6	45.7 ± 9.2	+ 8	<.001 (1.57)	.014 (0.87)	.742 (0.11)
TUG [s]	5.2 ± 0.4	5.0 ± 0.5	+ 4	5.3 ± 0.7	5.2 ± 0.5	+ 2	.098 (0.57)	.701 (0.13)	.521 (0.22)

Values are mean values ± standard deviations. Figures in brackets are effect sizes (Cohen’s *d*) with 0 ≤ *d* ≤ 0.49 indicating small, 0.50 ≤ *d* ≤ 0.79 medium, and *d* ≥ 0.80 large effects. ^a^A positive/negative percentage value indicates a performance improvement/decrement. *AT* Anterior, *BT-low* Balance training using a low level of task difficulty, *BT-high* Balance training using a high level of task difficulty, *CS* Composite score, *EC* Eyes closed, *EO* Eyes opened, *FI* Firm ground, *FO* Foam ground, *FRT* Functional Reach Test, *LL* Leg length, *OLS* One-legged stance, *PL* Posterolateral, *PM* Posteromedial, *TUG* Timed-Up-and-Go Test

### Static balance performance

**Fig. 3:**
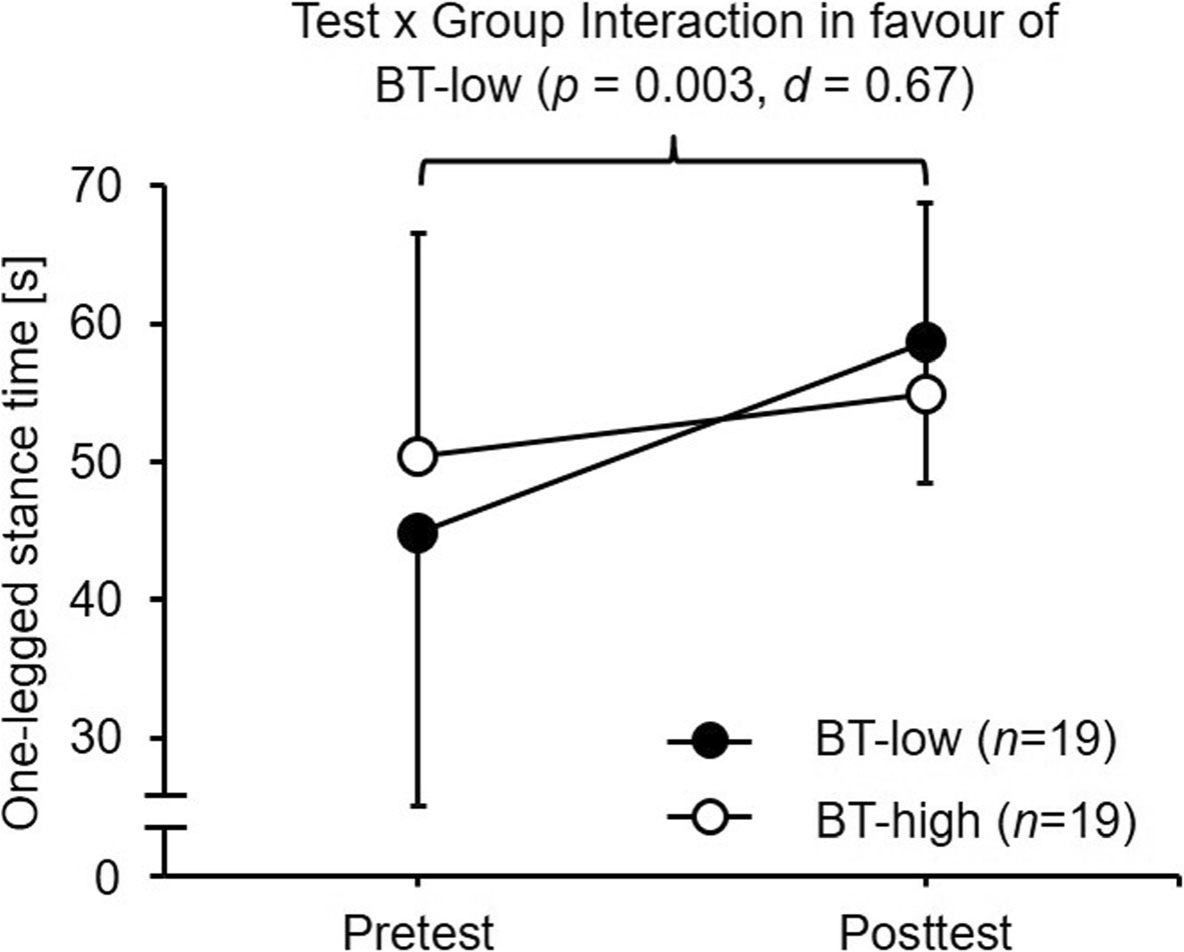
Performance changes (mean ± standard deviation) during the intervention period in static balance (i.e., one-legged stance time for the eyes opened, firm ground condition) for the BT-low compared to the BT-high group. BT-low = balance training using a low level of task difficulty; BT-high = balance training using a high level of task difficulty

Irrespective of stance condition, the analyses revealed statistically significant main effects of Test (13.391 ≤ *F*_1, 36_ ≤ 37.044, *p* ≤ 0.001, 1.22 ≤ *d* ≤ 2.03) (Table 2). Further, a tendency toward a significant Test × Group interaction was found for standing with eyes opened on firm surface (*F*_1, 74_ = 3.298, *p* = 0.078, *d* = 0.61). Post-hoc analyses revealed significant improvements from pre- to post-test in the BT-low (Δ + 31%, *p* = 0.003, *d* = 0.67) but not in the BT-high (Δ + 9%, *p* = 0.138, *d* = 0.30) group (Fig. 3). In addition, a significant main effect of Group (*F*_1, 36_ = 3.298, *p* = 0.037, *d* = 0.72) was observed for standing with eyes closed on firm surface. Furthermore, the number of participants that reached the maximal stance duration of 60 s increased from pre- to post-test in the BT-low (EO-FI: from 10 to 16; EC-FI: from 0 to 2; EO-FO: from 3 to 12) and in the BT-high (EO-FI: from 13 to 15; EC-FI: from 2 to 7; EO-FO: from 5 to 11).

### Dynamic balance performance

In terms of gait velocity, the analysis failed to detect significant main effects of Test and Group or a Test × Group interaction (Table 2).

### Proactive balance performance

**Fig. 4:**
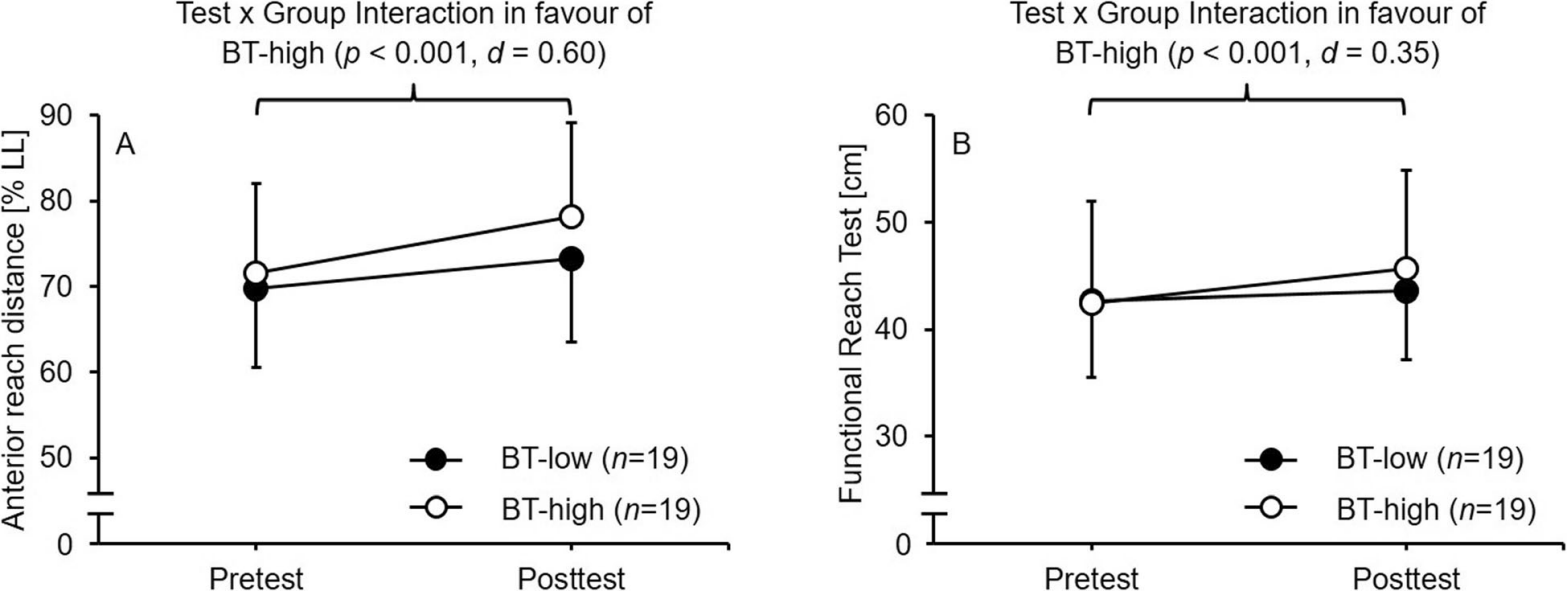
Performance changes (mean ± standard deviation) during the intervention period in proactive balance (i.e., A: anterior reach distance in the Y-Balance Test; B: Functional Reach Test distance) for the BT-low compared to the BT-high group. BT-low = balance training using a low level of task difficulty; BT-high = balance training using a high level of task difficulty; LL = leg length

For the YBT, the analyses indicated statistically significant main effects of Test for all reach directions (14.219 ≤ *F*_1, 36_ ≤ 34.767, *p* ≤ 0.001, 1.26 ≤ *d* ≤ 1.96) and the CS (*F*_1, 36_ = 41.342, *p* < 0.001, *d* = 2.15) (Table 2). Further, a tendency toward a significant Test × Group interaction was detected for the AT reach direction (*F*_1, 74_ = 3.388, *p* = 0.074, *d* = 0.61). Post-hoc analyses yielded significant enhancements over the course of training in the BT-high (Δ + 9%, *p* < 0.001, *d* = 0.60) and in the BT-low (Δ + 5%, *p* = 0.005, *d* = 0.36) group (Fig. 4a). The main effects of Group were not significant.

With regard to FRT, the analysis detected a significant main effect of Test (*F*_1, 36_ = 21.504, *p* < 0.001, *d* = 1.57) as well as a significant Test × Group interaction (*F*_1, 74_ = 6.663, *p* = 0.014, *d* = 0.87) (Table 2). Post-hoc analysis found that the participants in the BT-high group significantly increased their reach distance over the training period (Δ + 8%, *p* < 0.001, *d* = 0.35) while the participants in the BT-low group showed no significant changes (Δ + 2%, *p* = 0.161, *d* = 0.19) (Fig. 4b). The main effect of Group was not significant.

Concerning the TUG, the analysis showed a tendency toward a significant main effect of Test (*F*_1, 36_ = 2.894, *p* = 0.098, *d* = 0.57) (Table 2). The main effect of Group and the interaction effect of Test × Group were not significant.

## Discussion

We investigated the effects of BT using a low versus a high level of task difficulty on proxies of balance performance in healthy youth. The main findings of this study can be summarized as follows: (1) in both groups, all but one (i.e., 10-m gait velocity) measure of balance performance were significantly enhanced after the training period; (2) performance improvements in one parameter of static balance (i.e., OLS with eyes opened on firm ground) were small-sized for the BT-high and medium-sized for the BT-low group; (3) performance enhancements in some parameters of proactive balance (i.e., AT reach distance and FRT distance) were small- to medium-sized for the BT-high and small-sized for the BT-low group.

### Effects of balance training on measures of balance performance

In accordance with our hypothesis, we found that both exercise conditions resulted in enhanced balance performance. This finding corresponds with those from earlier studies investigating the impact of BT on measures of balance performance. For example, Granacher et al. [1] assigned high-school students to an intervention (BT) or an active control (P.E.) group. After 4 weeks of treatment (3 sessions per week), both groups improved their balance performance (i.e., reduced postural sway during one-legged stance) but the reductions were more pronounced in the BT compared to the P.E. group. Further, Heleno et al. [2] investigated the effect of 5 weeks (3 times per week) of BT additional to regular soccer training on proxies of static (30-s one-legged stance) and dynamic (modified Star Excursion Balance Test) balance in adolescent soccer players (mean age: ~ 15 years). In comparison to the active control group (i.e., soccer training only), the BT plus soccer training group exhibited greater enhancements of their balance performance (i.e., less postural sway and larger reach distance) than the soccer training only group. Lastly, Pau et al. [3] examined young volleyball players (mean age: 13.0 ± 0.2 years) that conducted 6 weeks (3 times per week) of BT in addition to regular volleyball training or regular volleyball training only. They found larger improvements in static balance performance (smaller postural sway areas during 20-s two-legged and 10-s one-legged stance) for the BT plus volleyball training group compared to the volleyball training only group. The results of the present study and those of the current literature [1–3] suggest that BT is a suitable exercise regimen to improve several proxies of balance performance in healthy youth.

### Effects of balance training difficulty on measures of balance performance

Partly in line with our hypothesis, we detected larger improvements in balance performance (i.e., AT reach distance and FRT distance) for the group that used a high compared to a low level of balance task difficulty during BT. There is empirical evidence [9–13] that balance performance can be challenged by the level of balance task difficulty. In this regard, Barbado Murillo et al. [11] assessed the effect of increasing difficulty in a standing balance task in young adults. As a result, the amplitude of postural sway significantly increased as the level of task difficulty changed from standing on a static surface over standing on a surface with medium followed by high instability. Further, Muehlbauer et al. [9] investigated young adults that performed the one-legged stance under various sensory conditions with an increased level of task difficulty. They found that altering the stance conditions from eyes opened on firm ground over eyes opened on foam ground to eyes closed on firm ground resulted in significant increases in postural sway. In addition, Donath and colleagues [12] examined young and older adults while they performed five standing tasks with increasing postural demand. Irrespective of the investigated age cohort, they reported significant increases in postural sway for the 30-s two-legged stance as the visual input (from eyes opened to closed) and the standing surface (from firm to foam) were changed. Lastly, Gebel et al. [13] studied the impact of graded task difficulty using a balance board with an adjustable pivot point to reduce the base of support diameter from 14 cm (level 1) to 4 cm (level 6) in adolescents. As the difficulty level changed from 1 to 6, the postural sway significantly increased.

Besides these findings from cross-sectional studies, there is additional evidence from one intervention study [20]. More precisely, Blasco et al. [20] determined the effect of BT with different stability conditions on balance performance in young adults. Participants were assigned to BT groups that trained on stable ground (corresponds to BT-low) or on unstable surfaces (corresponds to BT-high). Following 3 weeks of treatment (3 sessions per week), both groups improved their balance performance (i.e., YBT and FRT reach distance, one-legged stance time). But contrary to the present study, the improvements were not statistically different between groups. This discrepancy may be explained by the longer training period in the present study (i.e., 7 weeks) as compared to the study of Blasco and colleagues (i.e., 3 weeks) as it is assumed that longer training periods evoke larger adaptations.

What are likely explanations for our observation that participants in the BT-high group achieved partly larger improvements than those in the BT-low group? In addition to assessing behavioral measures (postural sway), the aforementioned cross-sectional studies also analyzed neuromuscular measures (muscle activity). All studies reported not only significant increases in variables of postural sway but also in parameters of muscle activation as a result of increased task difficulty. Enhanced muscular activity is achieved by recruiting additional motor units, increasing the frequency and/or improved synchronization of motor units [21]. Thus, regularly performing balance tasks of high as compared to low difficulty allows larger adaptive processes, which explains the larger improvements we observed in the BT-high compared to the BT-low group. Furthermore, BT-high may elicit larger changes in sensory integration and postural control strategy compared to BT-low. In this regard, van Dieen et al. [22] assessed balance performance during single leg stance in individuals learning to balance on an unstable device while also manipulating vestibular (i.e., galvanic vestibular stimulation) and visual (i.e., moving visual field) input. Participants initially increased proprioceptive input, but with practice upweighted visual and vestibular information indicating changes in the use of sensory information. Additionally, following 6 weeks of balance training on an unstable surface (i.e., slackline) Giboin et al. [23] reported decreased H-reflex amplitudes during stepping on a slackline indicating a more anticipatory/feedforward organization of postural control. Another explanation relates to the central nervous plane [24–27]. For example, Gebel et al. [24] used electroencephalographic analyses to examine the impact of increasing balance task difficulty on cortical activity in adolescents. They observed significant increases in the theta frequency band power and decreases in the alpha-2 frequency band power with increasing levels of task difficulty. The authors interpreted their finding as indicating enhanced attentional processes and/or error monitoring as well as increased sensory information processing due to increasing postural demands. This would also imply larger adaptive capacities, which would also explain the superior performance of the BT-high compared to the BT-low group.

## Conclusions

In the present study, we investigated the effects of BT conducted with a low versus a high level of balance task difficulty in adolescents. For both training regimens, we detected significant improvements in measures of static and proactive but not dynamic balance performance. Additionally, the enhancements in static balance were medium-sized for the BT-low and small-sized for the BT-high group. Further, the BT-low group showed small-sized and the BT-high group yielded small- to medium-sized improvements in proactive balance. Thus, it seems that BT-high is more effective for increasing measures of proactive balance, whereas BT-low is more effective for improving proxies of static balance.

## Data Availability

The data generated and analyzed during the present study are not publicly available due to ethical restrictions but are available from the corresponding author upon reasonable request.

## Abbreviations

ANOVA: Analysis of variance
AT: Anterior
BMI: Body mass index
BT: Balance training
BT-high: Balance training using a high level of task difficulty
BT-low: Balance training using a low level of task difficulty
CS: Composite score
EC: Eyes closed
EO: Eyes opened
FI: Firm ground
FO: Foam ground
FRT: Functional Reach Test
LL: Leg length
OLS: One-legged stance
PL: Posterolateral
PM: Posteromedial
TUG: Timed-Up-and-Go Test
